# Quality of Life in Obese Patients from a Multidisciplinary Bariatric Consultation: A Cross-Sectional Study Comparing to a Non-Bariatric Population and to the General Population

**DOI:** 10.3390/ijerph191912029

**Published:** 2022-09-23

**Authors:** Inês Rego de Figueiredo, Miguel Carvalho Vasques, Nelson Cunha, Diana Martins, José Silva-Nunes

**Affiliations:** 1 Multidisciplinary Unit for Bariatric and Metabolic Surgery, Centro Hospitalar Universitário Lisboa Central, 1069-166 Lisbon, Portugal; 2 Transplantation Unit, Centro Hospitalar Universitário Lisboa Central, 1069-166 Lisbon, Portugal; 3 NOVA Medical School/Faculdade de Ciencias Medicas, New University of Lisbon/Universidade Nova de Lisboa, 1169-056 Lisbon, Portugal; 4 Department of Endocrinology, Diabetes and Metabolism, Centro Hospitalar Universitário Lisboa Central, 1069-166 Lisbon, Portugal; 5 Health and Technology Research Center, Escola Superior de Tecnologia da Saúde de Lisboa, 1990-096 Lisbon, Portugal

**Keywords:** obesity, quality of life, EQ-5D-3L, bariatric quality of life index

## Abstract

Obesity is a chronic disease defined by a body mass index of ≥30 kg/m^2^, which can result in a decrease in quality of life (QoL). Our study aim was to assess the QoL of an obese population of bariatric surgery (BS) candidates, and to compare it to both that of a non-bariatric obese population (C) and that of the general population. This was a cross-sectional study using: (1) the EQ-5D-3L instrument: comparing BS with the C population and with the Portuguese general population; and (2) the Bariatric Quality of Life (BQL) Index: comparing the two groups of obese patients. We included 228 BS and 68 C obese patients. BS patients had higher BMI (44 ± 6 kg/m^2^ vs. 41 ± 6.5 kg/m^2^; *p* < 0.001), higher waist circumference (130 ± 13 cm vs. 123 ± 17 cm; *p* = 0.03), and higher total body fat mass (49.9 ± 6.7% vs. 45 ± 6.7%; *p* < 0.001). QoL as evaluated by EQ-5D-3L was similar, but the BQL index showed lower QoL in BS patients (40.9 ± 8.9 vs. 44.2 ± 11.2; *p* = 0.01). Compared to the Portuguese general population, BS patients had lower QoL (VAS: 55 ± 19 vs. 74.9; *p* < 0.001; index: 0.33 ± 0.2 vs. 0.76; *p* < 0.001). Despite higher adiposity in the BS group, QoL was similar between the groups by EQ-5D-3L. Nevertheless, there was a decrease in the QoL for the BS patients as determined using the BQL, a tool with higher sensitivity to bariatric patients.

## 1. Introduction

Obesity is a chronic relapsing disease that results from an imbalance in energy intake, with subsequent excessive fat accumulation in the body that presents a risk to health [1]. The World Health Organization (WHO) defines obesity through body mass index (BMI, which is derived by dividing a person’s weight by their height squared and expressed in units of kg/m^2^); when the BMI is ≥30 kg/m^2^, we consider obesity to be present [1]. Obesity has been defined as an epidemic disease, affecting over 13% of the world population in 2016, following a tripling of its prevalence from 1975 to 2016, with over 4 million people dying annually [1]. In Portugal, according to the 1st National Health Examination Survey, in 2015, 67.6% of the adult population was overweight and 28.7% was obese [2]. In 2018, 46,269 deaths by obesity-related diseases were reported in Portugal, representing 43% of annual deaths [3].

The obesity burden, however, not only implies an increase in mortality. It remains a major risk factor for noncommunicable diseases—the so-called obesity comorbidities. Among several others, obesity is a risk factor for cardiovascular conditions, type 2 diabetes, some cancers, and musculoskeletal disorders, which account for important disability [1]. Disability in obesity has been said to account for the loss of over 200,000 adjusted years [3], resulting in 3.8 more days per year of work absenteeism in the obese [4]. Furthermore, the obesity issue is accompanied by important economic load, with direct medical costs estimated at USD 260 billion in the United States [5] and up to EUR 1.2 billion in Portugal [3]. Indirect costs, such as those from missing work, can result in an additional EUR 238 million per year [4].

Obesity has also been associated with important psychiatric and social implications. Mental health issues and conditions are frequent in obese patients, the most frequent being depression, anxiety, personality disorders, and eating disorders [6,7,8]. Patient-reported outcomes (PROs), such as quality of life (QoL), are also affected by obesity [9,10]. These issues are as relevant as other noncommunicable diseases associated with obesity and should be addressed as such in an all-inclusive management approach to obesity [10].

A decrease in QoL due to obesity has been observed in adults and in children using a variety of health-related QoL instruments [11,12,13]. The decrease in QoL is inversely correlated with the increase in BMI [11,13]. Some studies report higher obesity-associated decreases in QoL among older patients and patients of female gender [12]. Furthermore, body image issues affect QoL, and different treatment approaches for obesity will have different results regarding QoL [14].

Obesity prevention, management, and treatment are, therefore, of extreme importance to control this worldwide epidemic. Bariatric surgery remains the most effective treatment for severe obesity, with up to 70% excess weight loss at 1 year [15,16]. Additionally, its effectiveness is also shown in PRO improvement, namely, body image and QoL [17,18,19].

Quality of life can be evaluated by specific questionnaire like tools. Several generic health-related QoL instruments have been used in obese patients in an attempt to cover aspects of physical disability and mental health [20], such as the EQ-5D tool [21] or the 36-Item Short Form Survey (SF-36) [22]. More disease-specific (obesity-specific) questionnaires have been developed; however, they fail to assess gastrointestinal symptoms and surgery-related issues [23,24,25]. Therefore, surgery-specific tools have been developed, such as the Bariatric Quality of Life (BQL) Index directed to patients undergoing bariatric surgery, in an attempt to grasp obesity- and surgery-related issues [26].

The aim in this study was to assess the quality of life of a cohort of Portuguese obese patients who were candidates for bariatric surgery, using both a generic health-related tool (EQ-5D-3L) and a treatment-specific tool (BQL). Additionally, we compared this cohort with one of non-bariatric obese patients, using both QoL tools, and with the Portuguese normative population values for the EQ-5D-3L tool.

## 2. Material and Methods

### 2.1. Study Design and Participants

The current study was a cross-sectional observational study performed at a Bariatric Surgery Unit, with obese patients who were candidates for bariatric surgery.

All patients (female or male, from 18 years old) admitted to the clinic were invited to participate in the study and included if agreement was given. Exclusion criteria, besides unwillingness to participate, were an inability to read and/or write and an inability to understand Portuguese.

All participants signed an informed consent, and their data were anonymized. The project had the approval of the Hospital’s Ethics Committee (INV 27).

### 2.2. Questionnaires

Participants filled in the QoL questionnaire EQ-5D-3L, validated for the Portuguese language [27,28], together with the BQL Index [26]. BQL is also a QoL tool, specifically developed for patients submitted to bariatric surgery, that is currently being translated to Portuguese. In this tool, lower scores mean worse QoL.

Further data were collected regarding demographics, anthropometric assessment (weight (kg) and height (cm) from which the BMI (kg/m^2^) was calculated, total body fat mass (%), visceral fat (%), and waist circumference (cm)), and obesity-associated comorbidities (hypertension, type 2 diabetes, dyslipidemia, sleep apnea, osteoarticular disease, depression/anxiety, chronic venous insufficiency of the lower limbs, and non-alcoholic fatty liver disease). Data were additionally collected regarding eating patterns as assessed by the psychologists and nutritionists of our team according to the medical history and food diary: sweet eater (as defined by the Dutch Sweet Eating Questionnaire [29]), volume eater (or hyperphagia—eating over the amount required for body size/composition [30]), snacking (eating small amounts of food frequently, instead of 3 substantial meals [31]), night eating (morning anorexia, evening hyperphagia of 25% of daily food intake and insomnia [32]), nibbling/picking (eating in an unplanned and repetitive way between meals and snacks [33]), emotional eating (eating in response to positive or negative emotions [34] assessed by the Emotional Eater Questionnaire [35]), compulsive eating (overeating even without hunger, can be associated with binge eating or bulimia [36]), or binge eating (defined by the Diagnostic and Statistical Manual of Mental Disorders, fifth edition [36] and assessed by the Eating Disorder Diagnostic Scale [37]). In addition, the motivation for surgery was collected (issues related with body image, health issues, mobility/functionality, and QoL improvement).

### 2.3. Data Analysis

The data were analyzed by comparing obese patients eligible for bariatric surgery to obese patients who either did not fulfill the criteria or did not wish to be submitted to surgery, considered as a control group. The former group of patients was recruited in the Bariatric Surgery Unit. The latter group was recruited in the Multidisciplinary Obesity Consultation from the Department of Endocrinology, Diabetes and Metabolism of our hospital; these patients also filled in both questionnaires (EQ-5D-3L and BQL), following signed informed consent. Their demographic and clinical data were also collected. Furthermore, a comparison was made for the EQ-5D-3L questionnaire between our sample population and the Portuguese normative values [28]. QoL by the EQ-5D-3L was assessed using a Visual Analogue Scale (VAS) and index score, as well as individual health profiles (mobility, self-care, usual activities, pain/discomfort, and anxiety/depression).

Parametric data are expressed as the mean (standard error of the mean, SEM), and nonparametric data are expressed as the median (interquartile range, IQR). We performed a *t* test, or Wilcoxon test, or ANOVA for parametric data comparisons, and chi square test for non-parametric data; a Spearman test was applied to assess correlations. 

Statistical analysis was performed using STATA (StataCorp. Stata statistical software: release 14. College Station, TX, USA: StataCorp LP). A *p* value of <0.05 was considered statistically significant.

## 3. Results

### 3.1. Participant Characteristics

Our sample included a total of 228 obese patients who were candidates for bariatric surgery (BS) and 68 obese controls (C). Their average age was 46 ± 11.7 years old, with a prevalence of the female gender (74%). Their BMI was, on average, 45.6 ± 6.5 kg/m^2^, with higher frequency of class III obesity (68%). Their average total body fat was 48.7 ± 7%, with 13.9 ± 4.6% visceral fat and 128.7 ± 14 cm waist circumference (Table 1).

BS patients were younger (45 ± 10 years old vs. 51 ± 15 years old; *p* < 0.001) and with higher female predominance (78% vs. 63%; *p* = 0.017). BS patients were heavier (121 ± 21.6 kg vs. 109 ± 21.9 kg; *p* < 0.001), resulting in higher BMI (44 ± 6 kg/m^2^ vs. 41 ± 6.5 kg/m^2^; *p* < 0.001). The large majority of BS patients presented class III obesity (72% vs. 53% in C patients; *p* < 0.001). Waist circumference was also higher in BS patients (130 ± 13 cm vs. 123 ± 17 cm; *p* = 0.03), along with total body fat mass (49.9 ± 6.7% vs. 45 ± 6.7%; *p* < 0.001). However, visceral fat was similar between the groups (14 ± 4.7% vs. 12 ± 3%; *p* = 0.1) (Table 1).

The most common obesity-related comorbidities were osteoarticular disease (68%), depression/anxiety (61%), and hypertension (56%). The comorbidities were overall similar between the two groups, with the exception of osteoarticular disease and non-alcoholic fatty liver disease, which were more frequent in the BS group (75% vs. 44%; *p* < 0.001 and 36% vs. 19%; *p* = 0.01, respectively) (Table 1).

Volume eating (68%), sweet eating (64%), and emotional eating (47%) were the most frequent eating patterns overall. Certain eating patterns were more frequent in the BS group, namely, sweet eaters (68% vs. 48%; *p* = 0.005), volume eaters (75% vs. 44%; *p* < 0.001), and emotional eaters (52% vs. 26%; *p* < 0.001). The remaining profiles were similar between the groups (snacking, night eating, nibbling/picking, compulsive, and binge eating) (Table 1).

### 3.2. Quality-of-Life Data

QoL as measured by the EQ-5D-3L was similar between the two groups of obese patients (VAS 55 ± 19 vs. 57 ± 25; *p* = 0.6 and index 0.33 ± 0.2 vs. 0.32 ± 0.3; *p* = 0.6). When assessed by EQ-5D-3L health profiles, only for the “usual activities” domain was a significant difference between groups observed, with a higher prevalence of level 2 for BS patients (57% vs. 35%; *p* = 0.003); the remaining health profiles (mobility, self-care, pain/discomfort, and anxiety/depression) were similar between the groups (Table 2).

Using the BQL scale, which was developed specifically for patients submitted to bariatric surgery, a significantly lower index in QoL was observed in BS patients (40.9 ± 8.9 vs. 44.2 ± 11.2; *p* = 0.01) (Table 2).

In comparison to the Portuguese normative values [28], QoL measured by the EQ-5D-3L was significantly lower in the BS population, as assessed by the VAS (55 ± 19 vs. 74.9; *p* < 0.001) and index score (0.33 ± 0.2 vs. 0.76; *p* < 0.001). This difference was also observed in all health profiles, with higher level 2 prevalence in BS patients: mobility (63% vs. 16.2%, *p* < 0.001), self-care (38% vs. 4.4%, *p* < 0.001), usual activities (57% vs. 13.9%, *p* < 0.001) pain/discomfort (71% vs. 40%, *p* < 0.001), and anxiety/depression (56% vs. 30.1%, *p* < 0.001) (Table 2).

## 4. Discussion

As reported by other authors, patients looking for a surgical solution to treat obesity were younger and there was a higher proportion of women [38,39,40] when compared with those who sought non-surgical interventions. The fact that older age (over 65 years) is still a limitative parameter for surgical eligibility in Portugal may explain that difference. A hypothetically higher perception of health-related issues among women could render them more prone to selecting more effective solutions, like surgery, explaining the higher proportion of women in the BS group. In fact, women tend to resort to bariatric surgery earlier, with less disease burden, resulting in fewer complications [40]. Also, women have stronger issues with body image, which also explains the more frequent use of surgery as a treatment for obesity [40]. Regarding anthropometrics, the BS group had higher body weight, BMI, body fat percentage, and waist circumference. Although both groups had a median BMI of over 40 kg/m^2^, and although there were patients in the C group who could be eligible for bariatric surgery, the differences in anthropometrics could be explained by the fact that patients with very high BMI (far beyond 40 kg/m^2^) are directed mainly to a bariatric solution.

Obesity-associated comorbidities were overall similar between the groups, but there was a higher prevalence of osteoarticular disease and liver disease in the BS group. Osteoarthritis symptoms and severity have been shown to correlate with higher BMI [41], while non-alcoholic fatty liver disease associates itself with higher central adiposity [42].

As stated, the most frequent eating patterns overall were sweet eating, volume eating, and emotional eating, but these were also more prevalent in the BS group when compared to the non-surgical group. Interestingly, studies in the literature report binge eating, night eating, and emotional eating as the most frequent in obese patients [43,44,45,46], rather than the ones we observed. We hypothesize that the difference between the groups could explain the higher severity of obesity in the BS cohort.

Using the EQ-5D-3L, a generic tool, there were no differences in QoL between the BS and C groups regarding the VAS and index evaluations. This is in contrast with previous studies that reported a lower QoL for patients with higher BMI. Although a significant difference in BMI was present between the BS and C patients, the mean BMI was too high in both groups and, consequently, obesity-related comorbidities and limitations were high in both. We hypothesize that the difference in BMI between groups was not sufficient to express a more significant decrease in QoL. However, differences in anthropometrics, and the consequent difference in the prevalence of osteoarticular disease [47], could be enough to justify the reported difference in the “usual activities” profile, with a higher frequency of difficulties reported in the BS group. 

Despite the lack of significant differences between groups in the EQ-5D-3L tool, we did observe a difference with the BQL tool, with BS patients presenting lower QoL when compared to the C group. This QoL tool, besides being obesity-specific, is also surgery-specific. This is important because it is more capable of detecting issues particular to obese patients and, in our study, of differentiating patients following two different obesity management directions. Additionally, the BQL tool has been described to be more sensitive in detecting changes in QoL following bariatric surgery [26]; this will be the object of a future study for our team.

Furthermore, we also observed a significant difference in QoL measured by the EQ-5D-3L when compared to the Portuguese normative values. BS patients had lower QoL when assessed by the index, VAS, and all health profiles, which is in accord with the current literature depiction of lower QoL in obese patients [11,12,13]. Using the EQ-5D-3L index value to calculate the loss of quality-adjusted life years (QALYs) derived from obesity, we observed an impact of −0.46 QALYs. Future works should also address how much of an increase in QALYs is induced by bariatric surgery and how close it can reach to the QALYs of the general population.

Our study has some limitations, specifically with the C group. This group did not homogenously constitute patients without eligibility for bariatric surgery, but rather included patients who were eligible but refused to be submitted to a surgical procedure. Although there were important demographic, anthropometric, and clinical differences between the groups, these could have been sorted out by a larger sample size. We also experienced some missing data, which led to a decrease in the sample size.

## 5. Conclusions

To conclude, our study shows that Portuguese obese patients who are candidates for bariatric surgery have lower QoL when compared to obese patients who are not eligible for or refuse bariatric surgery, as evaluated using a surgery-specific QoL tool, and when compared to the Portuguese general population, using the EQ-5D-3L.

Future work will include the validation of the BQL tool in Portuguese, as well as a follow-up on the BS patients to assess their QoL following surgery.

## Figures and Tables

**Table 1:** Demographic, anthropometric, and clinical data regarding both the BS and C groups; NS = non-significant.

	Study Groups			
**Demographics**	BS	C	Total	*p*
Age (years)	45 (10)	51 (15)	46 (11.7)	<0.001
Gender (%)				0.017
Female	177 (78)	43 (63)	220 (74)	
Male	51 (22)	25 (37)	76 (26)	
Total	228 (77)	68 (23)	296	
**Anthropometrics**				
Weight (Kg)	121 (21.6)	109 (21.9)	118 (22)	<0.001
Height (m)	1.65 (0.09)	1.63 (0.09)	1.6 (0.09)	NS
BMI (kg/m^2^)	44 (6)	41 (6.5)	45.6 (6.5)	<0.001
BMI category (%)				<0.001
Class I obesity	7 (3)	15 (22)	22 (7)	
Class II obesity	57 (25)	17 (25)	74 (25)	
Class III obesity	164 (72)	36 (53)	200 (68)	
Total body fat mass (%)	49.9 (6.7)	45 (6.7)	48.7 (7)	<0.001
Visceral fat (%)	14 (4.7)	12 (3)	13.9 (4.6)	NS
Waist circumference (cm)	130 (13)	123 (17)	128.7 (14)	0.03
**Comorbidities**				
Hypertension (%)	126 (56)	39 (59)	165 (56)	NS
Type 2 Diabetes (%)	43 (19)	17 (26)	60 (20)	NS
Dyslipidemia (%)	82 (36)	31 (47)	113 (38)	NS
Sleep Apnea (%)	62 (27)	18 (27)	80 (27)	NS
Osteoarticular disease (%)	171 (75)	29 (44)	200 (68)	<0.001
Depression/anxiety (%)	142 (62)	37 (56)	179 (61)	NS
Venous insufficiency (%)	106 (47)	22 (33)	128 (44)	0.06
Non-alcoholic fatty liver disease (%)	82 (36)	13 (19)	95 (32)	0.01
**Eating pattern**				
Sweet eater (%)	152 (68)	30 (48)	182 (64)	0.005
Volume eater (%)	167 (75)	27 (44)	194 (68)	<0.001
Snaking (%)	61 (27)	13 (21)	74 (26)	NS
Night eating (%)	23 (10)	6 (10)	29 (10)	NS
Nibbling/Picking (%)	18 (8)	1 (2)	19 (7)	0.07
Emotional eating (%)	117 (52)	16 (26)	133 (47)	<0.001
Compulsive eating (%)	35 (16)	5 (8)	40 (14)	0.1
Binge eating (%)	6 (3)	-	6 (2)	0.1

**Table 2:** QoL data from the EQ-5D-3L and BQL between the BS and C groups and between BS and the Portuguese general population.

	Study Groups			
Quality of Life	BS	C	Total	Portuguese General Population ^a^
BQL test ^b^	40.9 (8.9)	44.2 (11.2)	41.6 (9.6)	
EQ-5D-3L test				
**Health profiles**				
Mobility ^c^ (%)				
Level 1	76 (36)	26 (42)	102 (37)	83.3
Level 2	134 (63)	34 (55)	168 (61)	16.2
Level 3	2 (1)	2 (3)	4 (2)	0.5
Self-care ^c^ (%)				
Level 1	130 (61)	42 (69)	172 (63)	95.2
Level 2	81 (38)	18 (30)	99 (36)	4.4
Level 3	1 (1)	1 (1)	2 (1)	0.4
Usual activities ^b,c^ (%)				
Level 1	89 (42)	37 (60)	126 (46)	83.7
Level 2	120 (57)	22 (35)	142 (52)	13.9
Level 3	2 (1)	3 (5)	5 (2)	2.4
Pain/discomfort ^c^ (%)				
Level 1	34 (16)	15 (24)	49 (18)	55.3
Level 2	150 (71)	36 (58)	186 (68)	40
Level 3	28 (13)	11 (18)	39 (14)	4.7
Anxiety/depression ^c^ (%)				
Level 1	74 (35)	21 (35)	95 (35)	65.6
Level 2	120 (56)	30 (50)	150 (55)	30.1
Level 3	19 (9)	9 (15)	28 (10)	4.3
**EQ VAS ^c^**	55 (19)	57 (25)	56 (21)	74.9
**EQ Index ^c^**	0.33 (0.2)	0.32 (0.3)	0.33 (0.23)	0.76

^a^ Source: Ferreira et al. [28]; ^b^ *p* < 0.05 in BS vs. C; ^c^ *p* < 0.01 in BS vs. Portuguese general population.

## Data Availability

The data presented in this study are available on request from the corresponding author. The data are not publicly available due to ethical issues.

## References

[1] World Health Organization (2022). WHO European Regional Obesity Report 2022.

[2] Barreto M., Gaio V., Kislaya I., Antunes L., Rodrigues A.P., Silva A.C., Vargas P., Prokopenko T., Santos A.J., Namorado S. (2016). Estado de Saúde 1o Inquérito Nacional de Saúde Com Exame Físico INSEF.

[3] Borges M., Miguel L., Matias J., Lourenço F., Sousa R., Cardoso M., Costa J., Freitas P., Dias C., Gaio V. (2022). Custo e Carga Do Excesso de Peso e Da Obesidade Em Portugal.

[4] Destri K., Alves J., Gregório M.J., Dias S.S., Henriques A.R., Mendonça N., Canhão H., Rodrigues A.M. (2022). Obesity-Attributable Costs of Absenteeism among Working Adults in Portugal. BMC Public Health.

[5] Cawley J., Biener A., Meyerhoefer C., Ding Y., Zvenyach T., Gabriel Smolarz N.B., Ramasamy A. (2021). Direct Medical Costs of Obesity in the United States and the Most Populous States. J. Manag. Care Spec. Pharm..

[6] Jorm A.F., Korten A.E., Christensen H., Jacomb P.A., Rodgers B., Parslow R.A. (2003). Association of Obesity with Anxiety, Depression and Emotional Well-Being: A Community Survey. Aust. N. Z. J. Public Health.

[7] Rajan T.M., Menon V. (2017). Psychiatric Disorders and Obesity: A Review of Association Studies. J. Postgrad. Med..

[8] Malik S., Mitchell J.E., Engel S., Crosby R., Wonderlich S. (2014). Psychopathology in Bariatric Surgery Candidates: A Review of Studies Using Structured Diagnostic Interviews. Compr. Psychiatry.

[9] Taylor V.H., Forhan M., Vigod S.N., McIntyre R.S., Morrison K.M. (2013). The Impact of Obesity on Quality of Life. Best Pract. Res. Clin. Endocrinol. Metab..

[10] Vallis M. (2016). Quality of Life and Psychological Well-being in Obesity Management: Improving the Odds of Success by Managing Distress. Int. J. Clin. Pract..

[11] Pimenta F.B.C., Bertrand E., Mograbi D.C., Shinohara H., Landeira-Fernandez J. (2015). The Relationship between Obesity and Quality of Life in Brazilian Adults. Front. Psychol..

[12] Busutil R., Espallardo O., Torres A., Martínez-Galdeano L., Zozaya N., Hidalgo-Vega Á. (2017). The Impact of Obesity on Health-Related Quality of Life in Spain. Health Qual. Life Outcomes.

[13] Ramasamy S., Joseph M., Jiwanmall S.A., Kattula D., Nandyal M.B., Abraham V., Samarasam I., Paravathareddy S., Paul T.V., Rajaratnam S. (2020). Obesity Indicators and Health-Related Quality of Life—Insights from a Cohort of Morbidly Obese, Middle-Aged South Indian Women. Eur. Endocrinol..

[14] Yazdani N., Elahi N., Sharif F., Hosseini S.V., Ebadi A. (2020). The Comparison of Morbid Obesity Quality of Life and Body Image between Surgery and Other Treatments: A Case-Control Study. J. Educ. Health Promot..

[15] Nguyen N.T., Varela J.E. (2017). Bariatric Surgery for Obesity and Metabolic Disorders: State of the Art. Nat. Rev. Gastroenterol. Hepatol..

[16] Chang S.H., Stoll C.R.T., Song J., Varela J.E., Eagon C.J., Colditz G.A. (2014). The Effectiveness and Risks of Bariatric Surgery an Updated Systematic Review and Meta-Analysis, 2003–2012. JAMA Surg..

[17] Nickel F., Schmidt L., Bruckner T., Büchler M.W., Müller-Stich B.P., Fischer L. (2017). Influence of Bariatric Surgery on Quality of Life, Body Image, and General Self-Efficacy within 6 and 24 Months—A Prospective Cohort Study. Surg. Obes. Relat. Dis..

[18] Silva J.N., Vasconcelos H., Figueiredo-Braga M., Carneiro S. (2018). O Impacto Da Cirurgia Bariátrica Na Qualidade de Vida Dos Indivíduos Obesos: Um Estudo Português. Acta Med. Port..

[19] Raaijmakers L.C.H., Pouwels S., Thomassen S.E.M., Nienhuijs S.W. (2017). Quality of Life and Bariatric Surgery: A Systematic Review of Short- and Long-Term Results and Comparison with Community Norms. Eur. J. Clin. Nutr..

[20] Khan M.A., Richardson J., O’Brien P. (2012). The Effect of Obesity upon Health Related Quality of Life (HRQoL): A Comparison of the AQoL-8D and SF-36 Instruments. Farmeconomia Health Econ. Ther. Pathw..

[21] Kind P., Spilker B. (1996). The EuroQoL Instrument: An Index of Health—Related Quality of Life.

[22] Hays R.D., Sherbourne C.D., Mazel R. (1995). User’s Manual for the Medical Outcomes Study (MOS) Core Measures of Health-Related Quality of Life.

[23] Le Pen C., Lévy E., Loos F., Banzet M.N., Basdevant A. (1998). “Specific” Scale Compared with “Generic” Scale: A Double Measurement of the Quality of Life in a French Community Sample of Obese Subjects. J. Epidemiol. Community Health.

[24] Karlsson J., Taft C., Sjöström L., Torgerson J.S., Sullivan M. (2003). Psychosocial Functioning in the Obese before and after Weight Reduction: Construct Validity and Responsiveness of the Obesity-Related Problems Scale. Int. J. Obes. Relat. Metab. Disord..

[25] Kolotkin R.L., Crosby R.D., Kosloski K.D., Williams G.R. (2001). Development of a Brief Measure to Assess Quality of Life in Obesity. Obes. Res..

[26] Weiner S., Sauerland S., Fein M., Blanco R., Pomhoff I., Weiner R.A. (2005). The Bariatric Quality of Life (BQL) Index: A Measure of Well-Being in Obesity Surgery Patients. Obes. Surg..

[27] Ferreira L.N., Ferreira P.L., Pereira L.N., Oppe M. (2014). The Valuation of the EQ-5D in Portugal. Qual. Life Res..

[28] Ferreira L.N., Ferreira P.L., Pereira L.N., Oppe M. (2014). EQ-5D Portuguese Population Norms. Qual. Life Res..

[29] van den Heuvel M., Hörchner R., Wijtsma A., Bourhim N., Willemsen D., Mathus-Vliegen E.M.H. (2011). Sweet Eating: A Definition and the Development of the Dutch Sweet Eating Questionnaire. Obes. Surg..

[30] Heymsfield S.B., Avena N.M., Baier L., Brantley P., Bray G.A., Burnett L.C., Butler M.G., Driscoll D.J., Egli D., Elmquist J. (2014). Hyperphagia: Current Concepts and Future Directions Proceedings of the 2nd International Conference on Hyperphagia. Obesity.

[31] Chaplin K., Smith P.A. (2011). Definitions and perceptions of snacking. Curr. Curr. Top. Nutraceutical Res..

[32] Vinai P., Carpegna G., Vallauri P., Cardetti S., Ferrato N. (1955). The Night-Eating Syndrome; a Pattern of Food Intake among Certain Obese Patients. Am. J. Med..

[33] Conceição E.M., Crosby R., Mitchell J.E., Engel S.G., Wonderlich S.A., Simonich H.K., Peterson C.B., Crow S.J., le Grange D. (2013). Picking or Nibbling: Frequency and Associated Clinical Features in Bulimia Nervosa, Anorexia Nervosa, and Binge Eating Disorder. Int. J. Eat. Disord..

[34] Reichenberger J., Schnepper R., Arend A.-K., Blechert J. (2020). Emotional Eating in Healthy Individuals and Patients with an Eating Disorder: Evidence from Psychometric, Experimental and Naturalistic Studies. Proc. Nutr. Soc..

[35] Garaulet M., Canteras M., Morales E., López-Guimera G., Sánchez-Carracedo D., Corbalán-Tutau M.D. (2012). Validation of a Questionnaire on Emotional Eating for Use in Cases of Obesity: The Emotional Eater Questionnaire (EEQ). Nutr. Hosp..

[36] American Psychiatric Association (2013). Diagnostic and Statistical Manual of Mental Disorders.

[37] Stice E., Telch C.F., Rizvi S.L. (2000). Development and Validation of the Eating Disorder Diagnostic Scale: A Brief Self-Report Measure of Anorexia, Bulimia, and Binge-Eating Disorder. Psychol. Assess.

[38] Cooper A.J., Gupta S.R., Moustafa A.F., Chao A.M. (2021). Sex/Gender Differences in Obesity Prevalence, Comorbidities, and Treatment. Curr. Obes. Rep..

[39] da Silva P.T., Patias L.D., da Alvarez G.C., Kirsten V.R., Colpo E., de Moraes C.M.B. (2015). Profile of patients who seek the bariatric surgery. Arq. Bras. Cir. Dig..

[40] Kochkodan J., Telem D.A., Ghaferi A.A. (2018). Physiologic and Psychological Gender Differences in Bariatric Surgery. Surg. Endosc..

[41] Gløersen M., Steen Pettersen P., Neogi T., Jafarzadeh S.R., Vistnes M., Thudium C.S., Bay-Jensen A.C., Sexton J., Kvien T.K., Hammer H.B. (2022). Associations of Body Mass Index with Pain and the Mediating Role of Inflammatory Biomarkers in People with Hand Osteoarthritis. Arthritis Rheumatol..

[42] Pang Q., Zhang J.Y., Song S.D., Qu K., Xu X.S., Liu S.S., Liu C. (2015). Central Obesity and Nonalcoholic Fatty Liver Disease Risk after Adjusting for Body Mass Index. World J. Gastroenterol. WJG.

[43] Mccuen-Wurst C., Ruggieri M., Allison K.C. (2018). Disordered Eating and Obesity: Associations between Binge Eating-Disorder, Night-Eating Syndrome, and Weight-Related Co-Morbidities. Ann. N. Y. Acad. Sci..

[44] Konttinen H., van Strien T., Männistö S., Jousilahti P., Haukkala A. (2019). Depression, Emotional Eating and Long-Term Weight Changes: A Population-Based Prospective Study. Int. J. Behav. Nutr. Phys. Act..

[45] da Luz F.Q., Hay P., Touyz S., Sainsbury A. (2018). Obesity with Comorbid Eating Disorders: Associated Health Risks and Treatment Approaches. Nutrients.

[46] Nagata J.M., Garber A.K., Tabler J.L., Murray S.B., Bibbins-Domingo K. (2018). Prevalence and Correlates of Disordered Eating Behaviors Among Young Adults with Overweight or Obesity. J. Gen. Intern. Med..

[47] Marchesini G., Natale S., Tiraferri F., Tartaglia A., Moscatiello S., Marchesini Reggiani L., Villanova N., Forlani G., Melchionda N. (2003). The Burden of Obesity on Everyday Life: A Role for Osteoarticular and Respiratory Diseases. Diabetes Nutr. Metab..

